# Role of Galectin-3 in intervertebral disc degeneration: an experimental study

**DOI:** 10.1186/s12891-024-07382-5

**Published:** 2024-04-01

**Authors:** Jianjiang Li, Nianrong Han, Zhenqiang Liu, Akram Osman, Leilei Xu, Jing Song, Yang Xiao, Wei Hu

**Affiliations:** The Second Spine Department, The Fourth School of Clinical Medicine of Xinjiang Medical University, Urumqi, 830000 China

**Keywords:** Galectin 3, Intervertebral disc degeneration, Cartilage endplate degeneration

## Abstract

**Background:**

This study investigated the role of Galectin-3 in the degeneration of intervertebral disc cartilage.

**Methods:**

The patients who underwent lumbar spine surgery due to degenerative disc disease were recruited and divided into Modic I, Modic II, and Modic III; groups. HE staining was used to detect the pathological changes in endplates. The changes of Galectin-3, MMP3, Aggrecan, CCL3, and Col II were detected by immunohistochemistry, RT-PCR, and Western blot. MTT and flow cytometry were used to detect cartilage endplate cell proliferation, cell cycle, and apoptosis.

**Results:**

With the progression of degeneration (from Modic I to III), the chondrocytes and density of the cartilage endplate of the intervertebral disc decreased, and the collagen arrangement of the cartilage endplate of the intervertebral disc was broken and calcified. Meanwhile, the expressions of Aggrecan, Col II, Galectin-3, Aggrecan, and CCL3 gradually decreased. After treatment with Galectin-3 inhibitor GB1107, the proliferation of rat cartilage end plate cells was significantly reduced (*P* < 0.05). GB1107 (25 µmol/L) also significantly promoted the apoptosis of cartilage endplate cells (*P* < 0.05). Moreover, the percentage of cartilage endplate cells in the G1 phase was significantly higher, while that in the G2 and S phases was significantly lower (*P* < 0.05). Additionally, the mRNA and protein expression levels of MMP3, CCL3, and Aggrecan in rat cartilage end plate cells were lower than those in the control group.

**Conclusions:**

Galectin-3 decreases with the progression of the cartilage endplate degeneration of the intervertebral disc. Galectin-3 may affect intervertebral disc degeneration by regulating the degradation of the extracellular matrix.

**Supplementary Information:**

The online version contains supplementary material available at 10.1186/s12891-024-07382-5.

## Background

The intervertebral discs are composed of annulus fibrosus, nucleus pulposus, and cartilage endplates. The rupture of the annulus fibrosus and the metabolic imbalance of nucleus pulposus cells leads to a reduction in their ability to produce and maintain the extracellular matrix [1]. Intervertebral disc degeneration is a chronic progressive disease caused by a variety of factors and is characterized by the loss of nucleus pulposus cells, the degradation of the extracellular matrix, the changes in proteoglycans, loss of bound water molecules, and decreased tissue osmotic pressure [2]. Finally, these changes lead to changes in the mechanical action of the intervertebral disc, causing pain in specific areas. It has been shown that there is a high correlation between low back pain and the progression of intervertebral disc degeneration [3], and about 75-85% of elderly people worldwide suffer from low back pain caused by intervertebral disc degeneration [4, 5]. Intervertebral disc degeneration is related to many factors, including age, infection, injury, nutritional disorders, and inflammatory reactions. In recent years, as an important part of the intervertebral disc, the role of the intervertebral disc cartilage endplate has been extensively studied [6, 7]. The cartilage endplate of the intervertebral disc plays an important role in providing nutrition to the intervertebral disc and transmitting stress and has an important influence on the functional state and pathological changes of the intervertebral disc [8]. When the intervertebral disc cartilage endplate undergoes aging and degenerative changes, it will affect the biomechanics and nutrient supply status of the intervertebral disc, leading to the occurrence of intervertebral disc degeneration [9–11].

The cartilage endplate of the intervertebral disc is mainly composed of chondrocytes, type II collagen, proteoglycan, and other extracellular matrix. The main factors that trigger the degeneration of the intervertebral disc cartilage endplate include stress, inflammation, abnormal hormone secretion, and metabolic changes [12–15]. Studies have shown that the pathological changes of cartilage endplate degeneration are often accompanied by an imbalance of extracellular matrix synthesis and degradation, which in turn affects the functional integrity of the cartilage endplate and promotes cartilage endplate degeneration [16, 17]. Meanwhile, inflammatory mediators also play a promoting role in endplate degeneration [18].

Galectin-3 is a 29 to 35 kDa protein, belonging to the β-galactoside binding animal lectin family, and is widely expressed in various tissues [19]. Galectin-3 can act as a regulator of the interaction between cells and chondrocytes and the activity of matrix metalloproteinases (MMPs) [20]. In addition, Galectin-3 mainly regulates inflammatory factors and chemokines, thereby aggravating the inflammatory response of cartilage tissue and accelerating the destruction of cartilage [21]. Furthermore, the expression of Galectin-3 is upregulated in nucleus pulposus cells and inhibition of Galectin-3 can alleviate spinal cord injury and intervertebral disc degeneration [22]. It has also been suggested that Galectin-3 may have a protective effect on cells and tissues involved in intervertebral disc degeneration, limiting collagen damage and changes in biomechanical properties [23]. However, there is no study on the expression and role of Galectin-3 in the cartilage endplate of the intervertebral disc.

Here, we first observed the expression of Galectin-3 in clinical samples of cartilage endplate degeneration of intervertebral discs and then explored the role of Galectin-3 in cartilage endplate degeneration. Our findings may help to further understand the mechanism of Galectin-3 in the development of intervertebral disc cartilage endplate degeneration.

## Methods

### Subjects

**Fig. 1:**
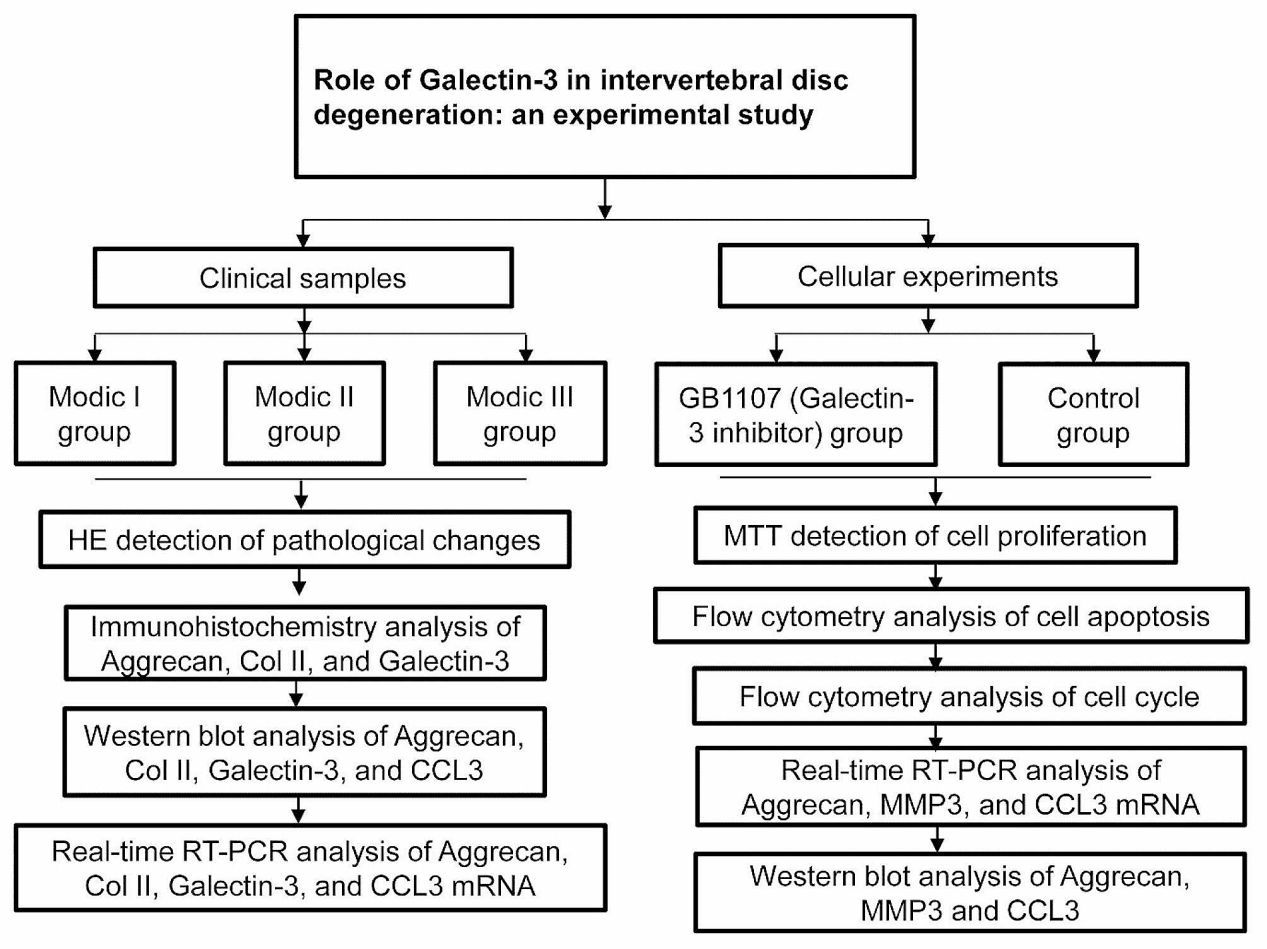
A flowchart depicting the sequence of events/tests performed in the study

This is an experimental study and the flowchart detailing the study process is shown in Fig. 1. Patients (*n* = 33) with lumbar spinal stenosis and lumbar spondylolisthesis who received minimally invasive transforaminal lumbar interbody fusion were enrolled in this study. There were 16 males and 17 females, with an average age of 46.1 ± 8.40 years. Inclusion criteria: (1) patients aged 30–65 years old; (2) patients who received lumbar MRI examination; (3) patients who had Modic changes in the lumbar endplate. Exclusion criteria: (1) patients with age < 30 or > 65 years; (2) patients with previous history of lumbar spine surgery; (3) patients combined with lumbar fractures, tumors, and infections; (4) patients combined with severe congenital abnormalities of the lumbar spine, complete or incomplete transitional vertebrae, or severe scoliosis. According to the different degrees of intervertebral disc degeneration, we further divided the patients into the Modic I group (*n* = 11), Modic II group (*n* = 11), and Modic III group (*n* = 11). The intervertebral disc cartilage endplate tissues were collected using spinal surgical tools such as reamers and scrapers during the lumbar surgery. Only the cartilage endplate was collected while leaving the subchondral bone intact. This study followed the Declaration of Helsinki and was approved by the ethics review board of the Xinjiang Uygur Autonomous Region Institute of Traditional Chinese Medicine. Written informed consent was obtained from every patient.

### Hematoxylin-eosin staining (HE) staining

The tissue specimens of the intervertebral disc cartilage endplate were fixed with 4% paraformaldehyde, and the decalcification agent was added to decalcify and soften the tissues. Then, paraffin embedding, and sectioning were performed. Finally, staining with hematoxylin and eosin staining, the histopathological changes were observed under a light microscope.

### Immunochemistry

After deparaffinization and hydration, the sections were treated with 3% H_2_O_2_ at room temperature for 10 min. After microwave heating in citrate buffer (pH = 6.0) for 10 min (92 ∼ 98 °C) for antigen retrieval, the sections were blocked with 5% calf serum at room temperature for 10 min. After that, the samples were incubated with 1:100 dilutions of anti-Aggrecan (13880-1-AP, Proteintech, China), anti-Col II (Collagen II) (Bs-0709R, Bioss, China), and anti-Galectin-3 (Ab209344, Abcam, USA) primary antibodies overnight at 4 °C, followed by incubation with 1:200 diluted HRP-conjugated secondary antibodies (ab205718, Abcam, USA) for 30 min at room temperature. After color development with DAB and counterstaining with hematoxylin, the samples were mounted and observed under a light microscope. The optical density of each protein was analyzed with IPP6.0 software. In detail, 11 samples of each group were used for quantification. To measure the optical density, three fields of each section were selected under 400 X magnification. The relative level of each protein was calculated by dividing the integrated optical density by the area.

### Cell culture

The rat cartilage endplate cells, which were isolated from the cartilage tissue of the intervertebral disc endplate, were purchased from Procell (Cat# CP-R152; Wuhan, China). They were cultured in a complete medium containing 10% fetal bovine serum at 37 °C with 5% CO_2_. The culture medium was changed every 2 days. When the confluency of cells was about 80%, the cells were passed at 1:3. Then, the P2 generation rat cartilage end plate cells were used for subsequent experiments.

### MTT assay

The rat cartilage endplate cells were seeded in a 96-well plate at 5 × 10^3^ cells/well and cultured overnight at 37 °C. Then, the cells were treated with Galectin-3 inhibitor GB1107 (1, 2.5, 5, 10, 25, 50 µmol/L) (Selleck, Houston, TX, USA) for 12 h, 24 h, and 48 h, respectively. Cell viability was detected by MTT assay. Briefly, 10 µL of MTT was added to each well and incubated at 37 °C for 4 h. After that, 150 µL of DMSO was added and incubated for 10 min. Finally, the result was measured at OD568 nm by a microplate reader.

### Apoptosis analysis by flow cytometry

The cells were seeded in a 6-well plate at 5 × 10^5^ cells/well and cultured overnight at 37 °C. Then, cells were treated with Galectin-3 inhibitor GB1107 (25 µmol/L) for 24 h. After that, the cells were collected and washed once with the binding buffer. The cells were stained with 7-AAD and Annexin V-APC from the apoptosis kit (AO2001-11 A-H, Tianjin Sungene Biotech Co., Ltd, China) in the dark for 10 min and then analyzed by flow cytometry (cytoFLEX, Beckman Coulter, USA).

### Cell cycle detection by flow cytometry

The cells were inoculated into a 6-well plate at 5 × 10^5^ cells/well and cultured overnight at 37 °C. Then, cells were treated with Galectin-3 inhibitor GB1107 (25 µmol/L) for 24 h. After that, the cells were collected and washed twice with PBS and fixed with 700 µL of pre-cooled 80% ethanol at 4 °C for 4 h. Then, the cells were stained with PI (50 ug/ml) at 4 °C in the dark for 30 min before flow cytometry detection.

### Real-time RT-PCR

Total RNAs were isolated from cartilage endplate tissues. Briefly, the tissues were first frozen in liquid nitrogen, then ground into powder, and lysed in TRIzol (Invitrogen, Carlsbad, CA, USA). Subsequently, a series of steps were carried out, including centrifugation, RNA isolation, ethanol precipitation, and determination of RNA purity and concentration using spectrophotometry. The isolated RNAs were then reverse-transcribed into cDNA with the TIANScript RT Kit (Tiangen, China). The reverse transcription reaction system (10 ul) was: 2ul 5×PrimeScript Buffer, 0.5ul PrimeScript RT Enzyme Mix I, 0.5ul Oligo (dT) 15 (15µM), 0.5ul random 6 mers (100 µM), Total RNA 500 ng, and RNase Free dH2O. Real-time RT-PCR primers were synthesized by Sangon Biotech (Shanghai, China), and the primer sequences are shown in Table 1.

**Table 1:** RT-PCR primers

Gene	Primer	Sequence (5’-3’)	PCR Products
Rat GAPDH	Forward	ACAGCAACAGGGTGGTGGAC	253 bp
	Reverse	TTTGAGGGTGCAGCGAACTT	
Rat Mmp3	Forward	GAATCCCCTGATGTCCTCGT	150 bp
	Reverse	TTTTCGCCAAAAGTGCCTGT	
Rat aggrecan	Forward	TGGACTTGTCTCAGGTTTC	295 bp
	Reverse	AGTTGGGGCAGTTATGGAT	
Rat CCL3	Forward	GCATTTAGTTCCAGCTCAGTGA	161 bp
	Reverse	GGACGGCAAATTCCACGAAA	
Homo GAPDH	Forward	TCAAGAAGGTGGTGAAGCAGG	115 bp
	Reverse	TCAAAGGTGGAGGAGTGGGT	
Homo aggrecan	Forward	TGAGCGGCAGCACTTTGAC	287 bp
	Reverse	TGAGTACAGGAGGCTTGAGG	
Homo CCL3	Forward	CATCACTTGCTGCTGACACG	192 bp
	Reverse	CGCTGACATATTTCTGGACCC	
Homo Galectin-3	Forward	CCCTGTGGATGCTTCAAACC	162 bp
	Reverse	GTAGGCCCCAGGATAGGAAG	
Homo Col II	Forward	TCCAGATGACCTTCCTACGC	209 bp
	Reverse	GGTATGTTTCGTGCAGCCAT	

The real-time RT-PCR was performed with SuperReal PreMix Plus (SYBR Green) (Tiangen, China). The reaction system included 0.8ul Forward primer 10 µM, 0.8ul Reverse primer 10 µM, 10ul 2XSYBR Select Master Mix, 6.4 ul RNase-free water, and 2ul cDNA template. The reaction procedure was 95 °C, 10 min; 95 °C, 15 s, 60 °C, 60 s, 95 °C, 15 s, 40 cycles; 60 °C 1 min, 95 °C, 15s. The 2^−ΔΔCt^ method was used for the quantitative calculation of results.

### Western blot

Total proteins were isolated from tissues and cells. Briefly, the cartilage endplate tissues were cut into small pieces, mixed with the lysis buffer (containing PMSF and phosphatase inhibitor) (Beyotime, Beijing, China), and homogenized in an automatic homogenizer. Subsequently, the cells and the homogenized tissues were subjected to lysis on ice. After 30 min, the lysate was centrifugated at 12,000 rpm for 5 min at 4 °C. Protein concentration in the supernatant was determined with the BCA protein concentration determination kit (Solarbio, China). After separation by electrophoresis, proteins were transferred to a membrane. After blocking, the membrane was probed with primary antibodies of rabbit polyclonal anti-Aggrecan (13880-1-AP, Tianjin Sungene Biotech Co., Ltd, China), rabbit polyclonal anti-Col II (Bs-0709R, Bioss, USA), rabbit monoclonal anti-Galectin-3 (Ab209344, Abcam, USA), rabbit polyclonal anti- C-C Motif Chemokine Ligand 3 (CCL3) (A7568, Affinity, China), rabbit monoclonal anti-MMP3 (ab52915, Abcam, USA), and anti-GAPDH (AB-P-R 001, Xianzhi Bio, Hangzhou, China). After washing, the membrane was incubated with the secondary antibody (Ab205718, Abcam, USA). After color development, the results were analyzed with Quantity-one software (Bio-Rad, USA). The gray value of the target protein to GAPDH was calculated, respectively.

### Statistical analysis

SPSS 15.0 software was used for data processing. Data are expressed as mean ± standard deviation (SD). One-way analysis of variance followed by the LDS method was used to compare the differences. A P value < 0.05 means that the difference is significant.

## Results

### Histopathological changes of cartilage endplate of intervertebral disc

**Fig. 2:**
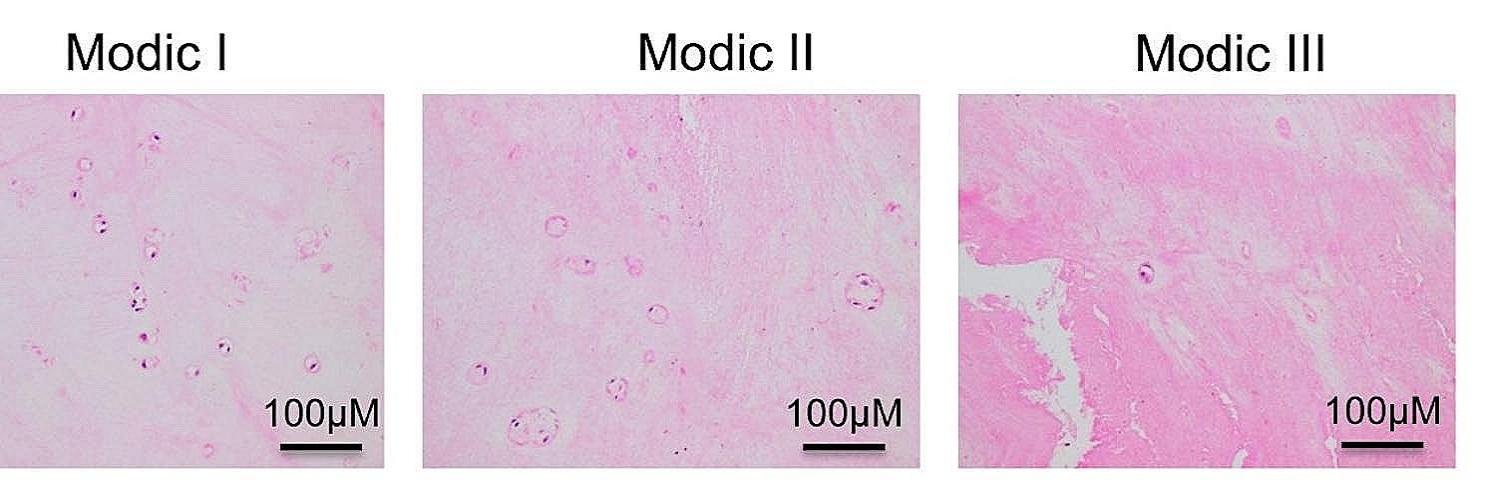
The pathological changes of the cartilage endplate tissue of the intervertebral disc. HE staining was performed to evaluate the pathological changes of the cartilage endplate tissue of the intervertebral disc from the Modic I group, the Modic II group, and the Modic III group. Scale bar: 100 µM

HE staining was used to detect the histopathological changes of the cartilage endplates of the intervertebral discs in the Modic I, Modic II, and Modic III groups (Fig. 2). The results showed that the collagen in the Modic I group was orderly arranged, accompanied by a small amount of chondrocyte apoptosis, but there was no obvious calcification. In the Modic II group, the number of chondrocytes and density of the intervertebral disc cartilage endplate decreased, and the collagen arrangement was irregular. Calcification was observed. In the Modic III group, the number and density of the cartilage endplate of the intervertebral disc were reduced; the collagen arrangement was broken; and the calcification was serious. These results indicated the degree of cartilage endplate damage aggravated as the disease got worse.

### Changes of Aggrecan, Col II, and Galectin-3 expression in the cartilage endplate of intervertebral disc

**Fig. 3:**
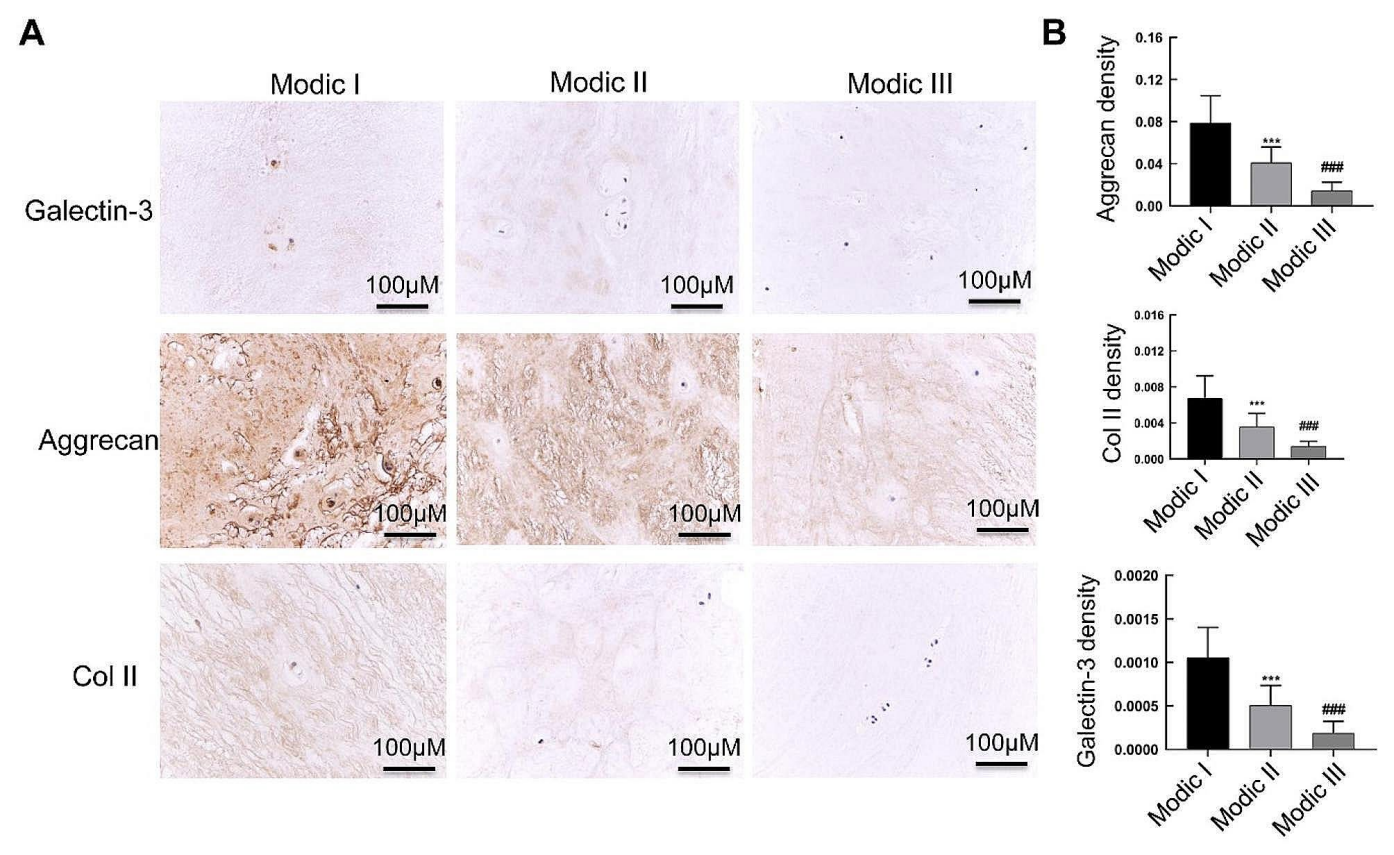
The changes of Aggrecan, Col II, and Galectin-3 expression in the cartilage endplate of the intervertebral disc. **A** The expression of Aggrecan, Col II, and Galectin-3 in the cartilage endplates of the intervertebral disc was detected by immunohistochemistry in Modic I, Modic II, and Modic III groups. Scale bar: 100 µM. **B** The relative expression levels of Aggrecan, Col II, and Galectin-3 in each group. Compared with Modic I group, ***P < 0.001

Immunohistochemical staining was used to detect the levels of Aggrecan, Col II, and Galectin-3 in the Modic I, Modic II, and Modic III-disc cartilage endplates (Fig. 3). The results showed that Galectin-3, Aggrecan, and Col II expressed at a high level in the Modic I group, while their expression in Modic II and Modic III gradually decreased (Fig. 3A). Statistically, the expression levels of Aggrecan, Col II, and Galectin-3 in the intervertebral disc cartilage endplates of the Modic III group were significantly lower than those in the Modic I and Modic II groups (Fig. 3B). Meanwhile, their levels in the Modic II group were significantly lower than those in the Modic I group.

**The mRNA levels of*Aggrecan, Col II, Galectin-3***, **and*CCL3*in the cartilage endplate tissue of the intervertebral disc**.

**Fig. 4:**
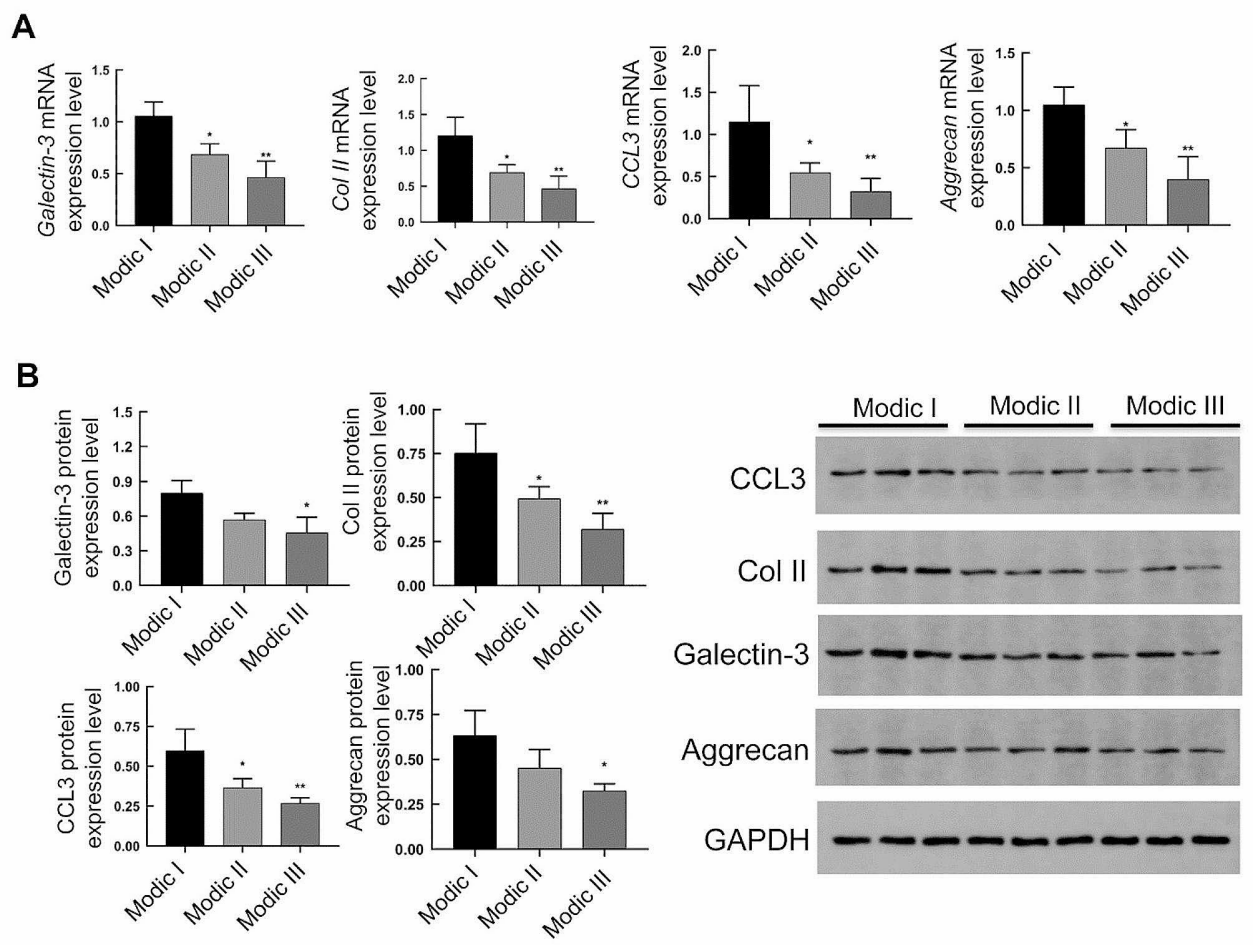
Levels of Aggrecan, Col II, Galectin-3, and CCL3 in the cartilage endplate tissue of the intervertebral disc in different groups. The mRNA and protein levels of Aggrecan, Col II, Galectin-3, and CCL3 in the cartilage endplate tissue of the intervertebral disc were analyzed with real-time RT-PCR (**A**) and Western blot (**B**), respectively. Compared with Modic I group, *P < 0.05, **P < 0.01

Real-time RT-PCR was used to detect the mRNA levels of *Aggrecan, Col II, Galectin-3*, and *CCL3* in the Modic I, Modic II, and Modic III intervertebral disc cartilage endplates (Fig. 4A). The results showed that *Aggrecan, Col II, Galectin-3*, and *CCL*3 mRNA levels were significantly lower in the Modic III and Modic II groups than in the Modic I group. However, there was no significant difference in their mRNA levels between Modic II and Modic III groups.

**Protein levels of Aggrecan, Col II, Galectin-3, and CCL3 in cartilage endplate tissue of the intervertebral disc**.

Western blot was used to detect the protein expressions of Aggrecan, Col II, Galectin-3, and CCL3 in the Modic I, Modic II, and Modic III groups of the intervertebral disc cartilage endplates. As shown in Fig. 4B, the expression of Galectin-3 and Aggrecan protein in the Modic III group was significantly lower than that in the Modic I group. Moreover, the expressions of Col II and CCL3 in the Modic III and Modic II groups were significantly lower than those in the Modic I group. However, there was no significant difference between the Modic II group and the Modic III group.

### Galectin-3 inhibitors cause decreased cell proliferation

**Fig. 5:**
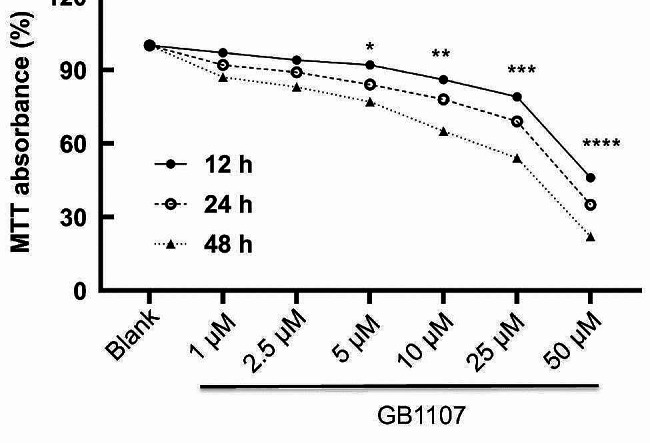
The effect of GB1107 on the proliferation of rat cartilage endplate cells. MTT assay was performed to detect the proliferation of rat cartilage endplate cells after treatment with GB1107. Compared with blank, *P < 0.05, **P < 0.01, ***P < 0.001, ****P < 0.0001

MTT was used to detect cell viability after treatment with different concentrations of Galectin-3 inhibitor GB1107 (1, 2.5, 5, 10, 25, 50 µmol/L) (Fig. 5). We found that the cell viability of the cartilage endplate cells decreased with treatment concentration and treatment time of GB1107. In detail, after 5–50 µmol/LGB1107 treatment, the cell viability at three different time points was significantly lower than that of the normal control group. The above results indicated that the cell proliferation capacity was negatively related to the Galectin-3 inhibitor concentration and treatment time.

### Galectin-3 inhibitor promotes apoptosis

**Fig. 6:**
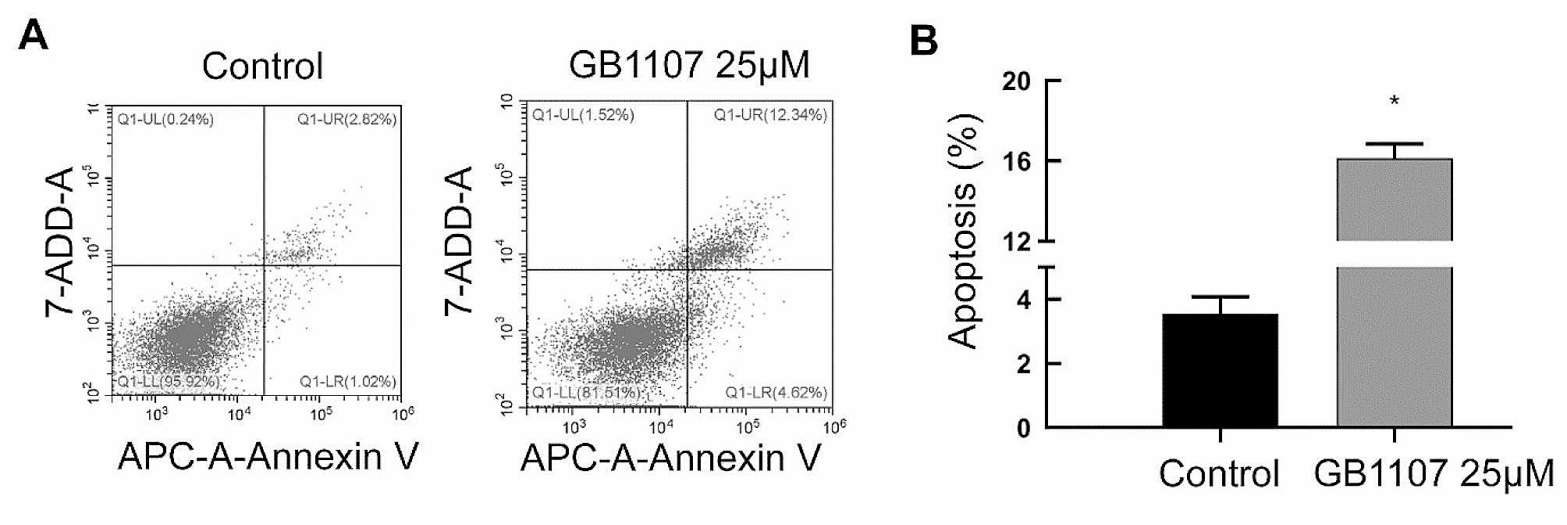
The effect of GB1107 on apoptosis of rat cartilage end plate cells. Cell apoptosis was detected with flow cytometry. Representative and quantitative flow cytometry results were shown in the left and right panels, respectively. Compared with the control group, *P < 0.05

Cell apoptosis was analyzed by flow cytometry. The results are shown in Fig. 6. Compared with the control, the Galectin-3 inhibitor GB1107 (25 µmol/L) significantly promoted apoptosis of rat cartilage end plate cells (*P* < 0.05).

### Galectin-3 inhibitor arrests the cell cycle in the G1 phase

**Fig. 7:**
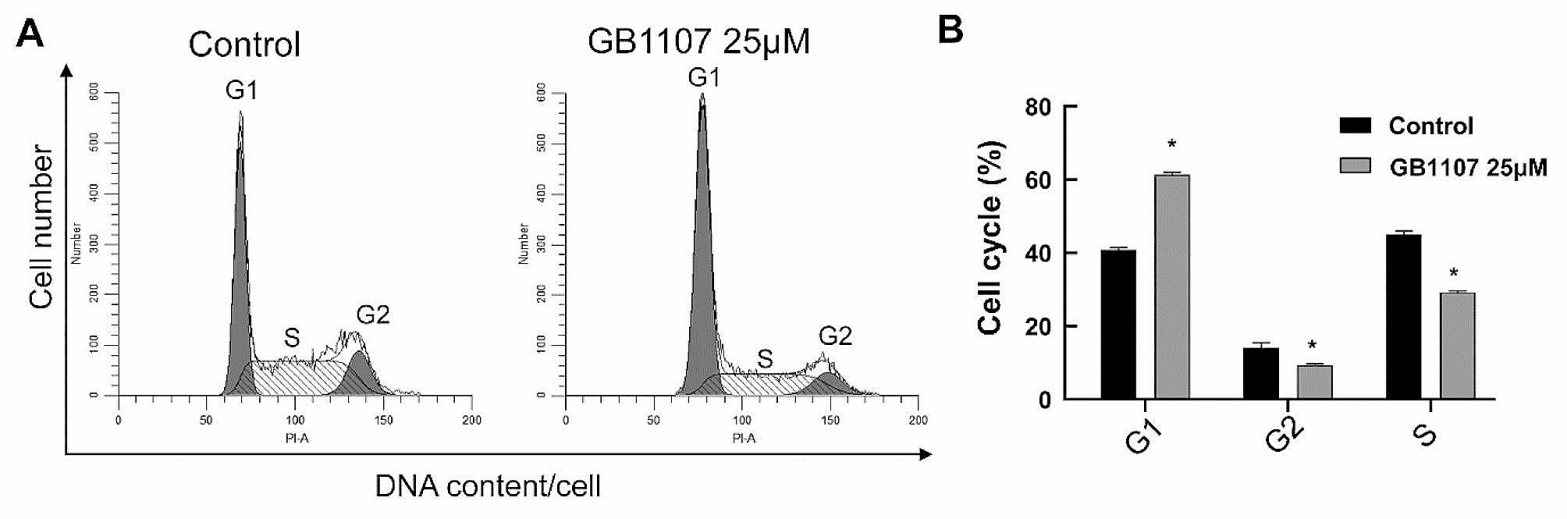
The effect of GB1107 on the cell cycle of rat cartilage end plate cells. The cell cycle was detected with flow cytometry. Representative and quantitative flow cytometry results were shown in the left and right panels, respectively. Compared with the control group, *P < 0.05

To investigate whether GB1107-induced apoptosis is related to cell cycle arrest, flow cytometry was used to detect the cell cycle distribution of rat cartilage endplate cells (Fig. 7). After Galectin-3 inhibitor treatment, the percentage of rat cartilage endplate cells in the G1 phase was significantly higher than that of the normal control group, while those in the G2 and S phases were significantly lower than those in the normal control group. Thus, GB1107 arrests the cell cycle in the G1 phase.

### Galectin 3 inhibitor suppresses the expression of MMP3, Aggrecan, and CCL3 in rat cartilage end plate cells

**Fig. 8:**
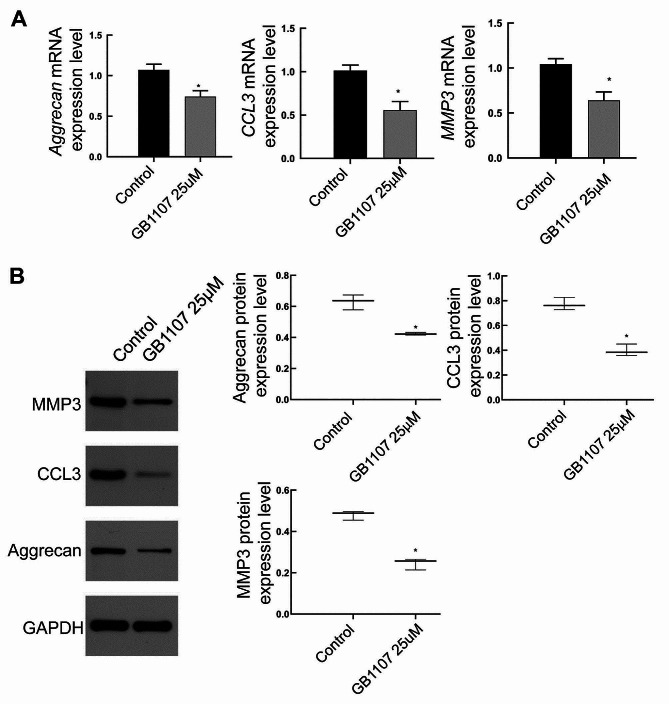
The mRNA and protein levels of MMP3, CCL3, and Aggrecan in cartilage end plate cells. The mRNA and protein levels of MMP3, CCL3, and Aggrecan in cartilage end plate cells were detected with real-time RT-PCR (**A**) and Western blot (**B**), respectively. Compared with the control group, *P < 0.05

Real-time RT-PCR results showed that after treatment with Galectin-3 inhibitor, the mRNA levels of *MMP3, CCL3*, and *Aggrecan* in rat cartilage end plate cells were significantly lower than those in the control group (Fig. 8A). Consistently, Western blot results also showed that treatment with Galectin-3 inhibitors significantly reduced the protein expression of MMP3, CCL3, and Aggrecan in rat cartilage endplate cells (Fig. 8B).

## Discussion

Intervertebral disc degeneration is the physiological and pathological process of a series of degenerative diseases of the intervertebral disc caused by the natural degeneration of the intervertebral disc and various risk factors. Intervertebral disc degeneration may be triggered by an imbalance in extracellular matrix synthesis and degradation caused by various pathogenic factors [24]. The pathogenic factors of intervertebral disc degeneration include stress, injury, inflammation, aging, bacterial infection, nutritional disorders, and tumors [12, 13]. Excessive mechanical load damages DNA, promotes cellular aging, reduces the secretion of synthetic metabolic factors such as transforming growth factor p and insulin-like growth factor, and inhibits the synthesis of extracellular matrix [25, 26]. The mitochondrion can be damaged by inflammatory factors produced by nucleus pulposus cells, fibroblast-like cells, and immune cells. This results in insufficient energy supply to the intervertebral disc tissue while promoting the synthesis of extracellular matrix-degrading enzymes, leading to the degradation of the extracellular matrix and causing an imbalance in extracellular matrix metabolism [27, 28]. Furthermore, excessive mechanical load and inflammatory stimulation can induce oxidative stress, generate a large amount of reactive oxygen species, further accelerate cellular aging, and induce cell apoptosis [29, 30]. The endplate is an important part of the intervertebral disc. It plays an important role in maintaining the normal shape of the vertebrae, protecting the vertebrae from pressure, and absorbing static pressure. Meanwhile, the endplate is also the main bearer of the osmotic transport capacity in the vertebral body and the nutrition supply pathway [6]. Studies have shown that cartilage endplate damage is directly related to cartilage endplate degeneration [31, 32]. However, the underlying mechanism is still not clear.

Besides endplate injuries, another manifestation of endplate pathology is the presence of Modic changes. Research on Modic changes primarily focuses on the lumbar spine. Modic changes are closely associated with intervertebral disc degeneration and lower back pain, and they are independent risk factors for severe lower back pain episodes. The size of Modic changes positively correlates with the severity of lower back pain. The Pfirrmann grading system is based on the structure of the nucleus pulposus, the boundaries of the nucleus pulposus and annulus fibrosus, the signal intensity of the nucleus pulposus, and the height of the intervertebral discs. This study primarily investigated cartilaginous endplate degeneration. Therefore, we used the Modic classification rather than the Pfirrmann classification. Our results showed that Modic type I, II, and III changes had a certain reduction in the number of chondrocytes and a disordered arrangement of collagen. Meanwhile, the mRNA and protein levels of Aggrecan, Col II, Galectin-3, and CCL3 also decreased accordingly, with the highest level in Modic I type and the lowest in Modic III type. This is consistent with previous findings, which have shown that Aggrecan and Col II, as the main components of the extracellular matrix, decreased with the increase of the degree of degeneration, eventually destroying the structure of the cartilage endplate and leading to the degeneration of the cartilage endplate [33]. Moreover, Galectin-3 can affect the inflammatory response by regulating the expression of the *CCL3* gene, thereby interfering with the progression of cartilage destruction [21].

Studies have shown that Galectin-3 can induce and regulate the secretion of inflammatory factors, and then participate in the generation and development of arthritis [19, 34, 35]. Galectin-3 can also inhibit the apoptosis of joints and hypertrophic chondrocytes and promote cell proliferation [20, 36–39]. Narjès Hafsia et al. found that compared with wild-type mice, the damage of chondrocytes in Galectin-3 knockout mice was more serious [40]. It is reported that after the cartilage endplate was damaged, the level of pro-inflammatory cytokines and the activity of matrix-degrading enzymes increased, while the activity of proteoglycan in the extracellular matrix of cartilage decreased, which in turn induced the degradation of the extracellular matrix and the destruction of the cartilage endplate and promotes degeneration of the endplate [41]. Although Galectin-3 has been extensively studied, its regulation of proliferation, apoptosis, and cell cycle in endplate cells has not yet been studied. In this study, by treating end plate cells with a Galectin-3 inhibitor, we explored the regulation of Galectin-3 on the proliferation and apoptosis of end plate cells. The results showed that Galectin-3 inhibitor significantly inhibited cell proliferation and apoptosis, which is consistent with the study of Arad et al. [42]. In addition, studies have found that through in vivo injection, Galectin-3 causes degeneration and destruction of cartilage in the knee joint of animal models [16, 43]. These results indicate that Galectin-3 inhibits cell activity and promotes apoptosis of chondrocytes, which is inconsistent with our results. This may be caused by the different cell types used. Further studies are needed to validate these results.

The cell cycle has three phases of G0/G1 phase, the S phase, and the G2/M phase [44]. In the cells treated with Galectin-3 inhibitor, we observed that most of the cells were in the G1 phase, resulting in a significant decrease in the number of cell populations in the S and G2 phases. These results imply that the Galectin-3 inhibitor may play a role in the G1/S phase checkpoint, leading to a decrease in DNA synthesis. In addition, the cell cycle is regulated by many signaling pathways, including the p53 signaling pathway, p21 signaling pathway, p27 signaling pathway, and PI3K/Akt signaling pathway [45, 46]. Feng et al. [47] found that under stress, the activated p53 signaling pathway regulated the premature aging of cartilage endplate cells. In addition, it is reported that the apoptosis of cartilage endplate cells was achieved by activating the p38 and MAPK signaling pathways and inhibiting the PI3K/Akt signaling pathway [37]. The specific mechanism underlying the role of Galectin-3 in the cell cycle remains to be further studied.

Aggrecan is a type of proteoglycan, which is generally aggregated in the cartilage endplate cell matrix in the form of a polymer, providing its permeability characteristics. It can also help intervertebral discs resist mechanical compression loads transmitted along the spine. With age, intervertebral discs dehydrate due to the loss of Aggrecan, and the degenerative process is further enhanced by repeated injuries, transforming the intervertebral discs into a degenerative state [16]. Our results showed that after inhibiting Galectin-3, the expression of Aggrecan decreased, which may result in a decrease in cell support. MMP-3 is the main member of the matrix metalloproteinase family. A large number of studies have shown that overexpression of MMP-3 can cause the degradation of the extracellular matrix, leading to degeneration of the intervertebral disc [48, 49]. However, it has also been shown that MMP3 can degrade proteoglycan aggregates and various matrix components within the intervertebral disc, reducing the dehydration effect of the nucleus pulposus and playing a protective role [50]. Here, we found that after inhibiting Galectin-3, the mRNA and protein levels of MMP3 in rat cartilage endplate cells decreased, suggesting that Galectin-3 may play a role in intervertebral disc degeneration by regulating the degradation of extracellular matrix in cartilage endplate cells and the expression levels of Aggrecan. CCL3, also known as the macrophage inflammatory protein, has the function of recruiting macrophages and plays an important role in inflammatory and immune responses [51]. The number of macrophages is positively correlated with the degree of intervertebral disc degeneration [4]. We found that after inhibiting Galectin-3, the mRNA and protein expression levels of CCL3 in rat cartilage endplate cells decreased. These indicate that Galectin-3 can affect the expression of CCL3, may play a role in the inflammatory cell infiltration of the intervertebral disc cartilage endplate, and affect the occurrence of intervertebral disc degeneration.

This study has several limitations. Firstly, it did not consider Pfirrmann grading as part of the research method. Secondly, the study primarily focused on studying the patient population and did not include a control group, potentially introducing biases. Thirdly, we did not conduct specific inhibition experiments using si-RNA to validate our conclusion further, nor did we evaluate the direct effect of Galectin-3 on endplate cells by using recombinant human Galectin-3. Further studies are warranted.

## Conclusions

In summary, with the progression of intervertebral disc degeneration, the expression of Galectin-3 in clinical tissues decreases, which may further promote intervertebral disc degeneration. Through in vitro experiments, we also verified that low-expressed Galectin-3 could regulate cartilage endplate cell proliferation, apoptosis, and cell cycle, thus regulating intervertebral disc degeneration. Our findings may contribute to a better understanding of the role of Galectin-3 in the development of degeneration in the intervertebral disc cartilage endplate. Additionally, the findings of this study could offer valuable evidence for preventing and treating degenerative conditions in intervertebral discs.

### Electronic supplementary material

Below is the link to the electronic supplementary material.

Supplementary Material 1

## Data Availability

The datasets used and/or analyzed during the current study are available from the corresponding author upon reasonable request.

## Abbreviations

MMPs: matrix metalloproteinases
HE: Hematoxylin-eosin staining
