# Supplementary material for: Role of Galectin-3 in intervertebral disc degeneration: an experimental study
Source: BMC Musculoskelet Disord. 2024 Apr 1;25:249. doi: 10.1186/s12891-024-07382-5 (PMC10983641; doi:10.1186/s12891-024-07382-5)
Supplement: Supplementary file 1 — Supplementary Material 1 [file 12891_2024_7382_MOESM1_ESM.pptx]

## Slide 1
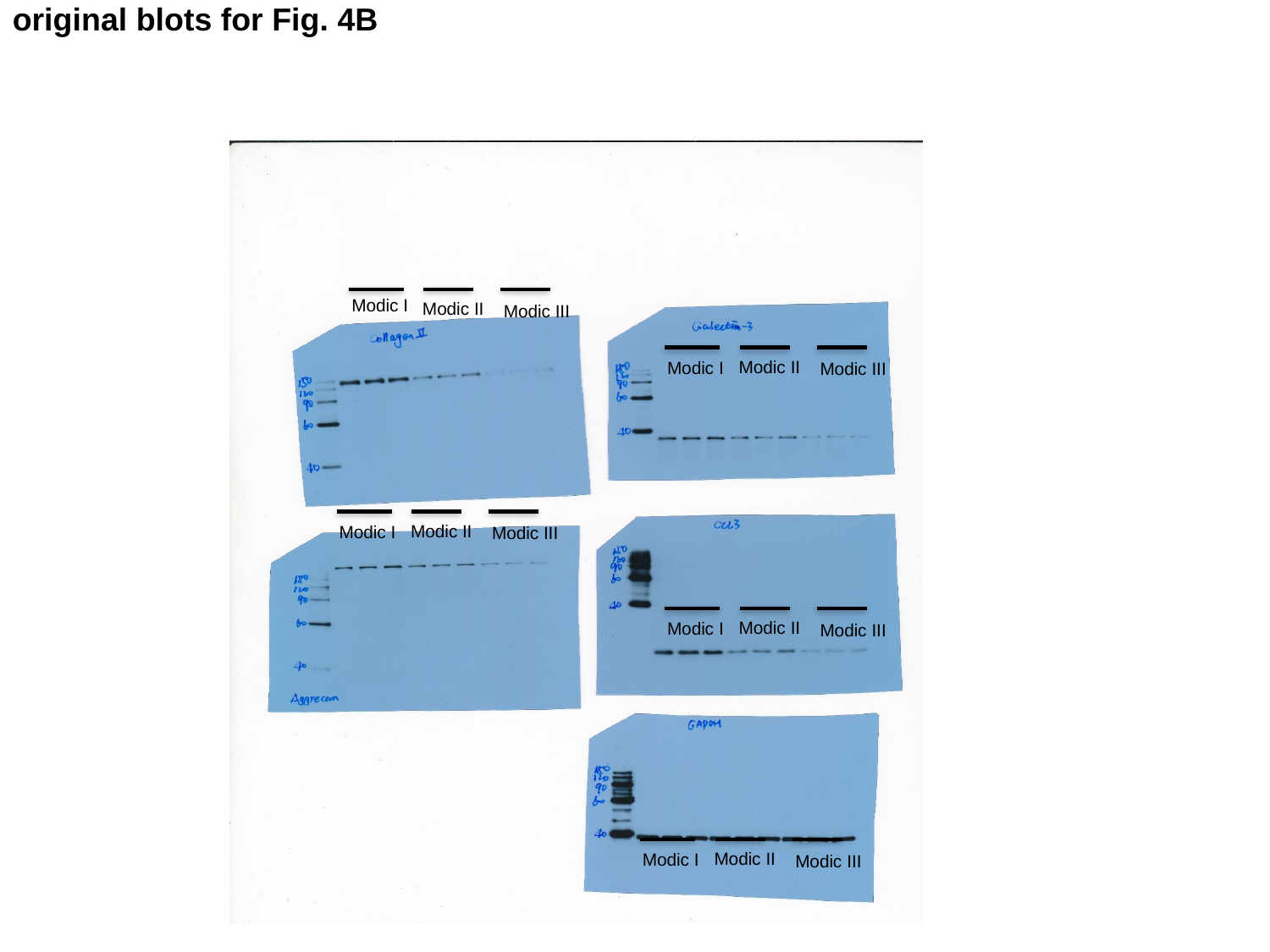

original blots for Fig. 4B
Modic I
Modic II
Modic III
Modic II
Modic I
Modic III
Modic II
Modic I
Modic III
Modic II
Modic I
Modic III
Modic II
Modic I
Modic III

## Slide 2
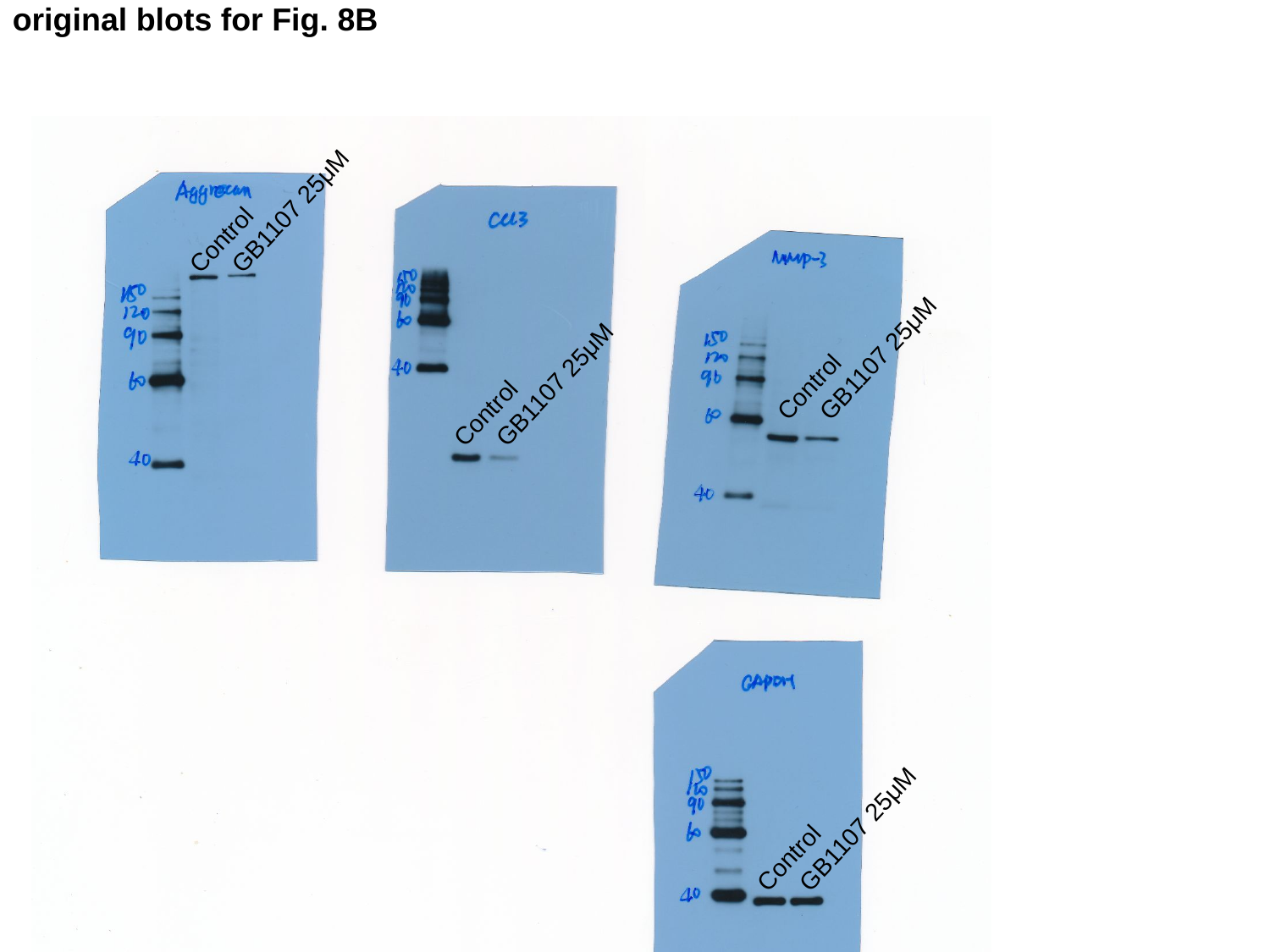

original blots for Fig. 8B
GB1107 25μM
Control
GB1107 25μM
GB1107 25μM
Control
Control
GB1107 25μM
Control
